# Calcium impacts carbon and nitrogen balance in the filamentous cyanobacterium *Anabaena* sp. PCC 7120

**DOI:** 10.1093/jxb/erw112

**Published:** 2016-03-24

**Authors:** Julia Walter, Fiona Lynch, Natalia Battchikova, Eva-Mari Aro, Peter J. Gollan

**Affiliations:** Department of Biochemistry, Molecular Plant Biology, University of Turku, FI-20014 Turku, Finland

**Keywords:** Anabaena, bicarbonate, calcium, cmpA, cyanobacteria, nirA, nitrogen, transcriptomics.

## Abstract

Calcium is integral to the perception, communication and adjustment of cellular responses to environmental changes. However, the role of Ca^2+^ in fine-tuning cellular responses of wild-type cyanobacteria under favourable growth conditions has not been examined. In this study, extracellular Ca^2+^ has been altered, and changes in the whole transcriptome of *Anabaena* sp. PCC 7120 have been evaluated under conditions replete of carbon and combined nitrogen. Ca^2+^ induced differential expression of many genes driving primary cellular metabolism, with transcriptional regulation of carbon- and nitrogen-related processes responding with opposing trends. However, physiological effects of these transcriptional responses on biomass accumulation, biomass composition, and photosynthetic activity over the 24h period following Ca^2+^ adjustment were found to be minor. It is well known that intracellular carbon:nitrogen balance is integral to optimal cell growth and that Ca^2+^ plays an important role in the response of heterocystous cyanobacteria to combined-nitrogen deprivation. This work adds to the current knowledge by demonstrating a signalling role of Ca^2+^ for making sensitive transcriptional adjustments required for optimal growth under non-limiting conditions.

## Highlight

Calcium affects the primary cellular metabolism of *Anabaena* under conditions replete in both combined-nitrogen and inorganic carbon. Opposite transcriptome responses to calcium treatments occur for nitrogen- and carbon-related processes.

## Introduction

Cyanobacteria form a diverse clade of highly specialized bacteria able to perform oxygenic photosynthesis and thus are proclaimed to be the evolutionary ancestors of the chloroplasts of photoautotrophic eukaryotes (Douglas, 1998; Falcon *et al.*, 2010). They comprise five different groups based on their morphology and metabolic pathways (Rippka *et al.*, 1979). For instance, there are unicellular cyanobacteria, such as the model organism *Synechocystis* sp. PCC 6803 (Kaneko and Tabata, 1997), and multicellular (filamentous) cyanobacteria, of which *Anabaena* sp. PCC 7120 (hereafter designated as *Anabaena*) is one of the common models (Kaneko *et al.*, 2001). *Anabaena* also represents the heterocystous cyanobacteria that can fix atmospheric nitrogen in conditions lacking combined nitrogen (Wolk, 1996; Flores and Herrero, 2005; Zhang *et al.*, 2006).

Calcium (Ca^2+^) is well known to play an essential role as second messenger and signalling molecule in all living organisms (Clapham, 1995). In plants, Ca^2+^ pulses of specific magnitude, duration, frequency, and source are called Ca^2+^ ‘signatures’ and are triggered in response to many different environmental stimuli in order to activate response mechanisms (Knight M. *et al.*, 1991, 1992; Knight H. *et al.*, 1996, 1997; Knight and Knight, 2000). However, besides the calcium signals, the cytosolic free Ca^2+^ concentration ([Ca^2+^]_i_) is tightly regulated by Ca^2+^-binding proteins, Ca^2+^ pumps and other transporters to maintain low [Ca^2+^]_i_ levels of about 100nM in order to avoid toxic calcium phosphate precipitation within the cell.

Recently, an important role of Ca^2+^ sequestration and signalling has been highlighted in regulating the activities of chloroplasts (Stael *et al.*, 2011, 2012a, b; Rocha and Vothknecht, 2012; Nomura and Shiina, 2014). Meanwhile, in cyanobacteria, the chloroplast ancestors, information concerning Ca^2+^-regulated processes is still rather scarce. Cyanobacteria have been shown to use Ca^2+^ signalling under various environmental stimuli (Torrecilla *et al.*, 2000, 2001; Barrán-Berdón *et al.*, 2011), but so far only one cyanobacteria-specific Ca^2+^-binding protein (Ccbp in *Anabaena*) has been characterized (Zhao *et al.*, 2005; Shi *et al.*, 2006; Hu *et al.*, 2011; Dominguez *et al.*, 2015). Ccbp is involved in the signalling cascade resulting in the formation of nitrogen-fixing heterocysts (Flores and Herrero, 2005). The concept of Ca^2+^ being involved in atmospheric nitrogen fixation was reported as early as the 1930s by Burk *et al.* (1934), and was supported several times over the following decades (Allison *et al.*, 1936; Allen and Arnon, 1955; Mishra *et al.*, 1985). It is now known that Ca^2+^ controls this process in *Anabaena* through the negative regulation of Ccbp by HetR and the transcription factor NtcA, by which the release of Ca^2+^ induces the differentiation of vegetative cells into nitrogen-fixing heterocysts. The process of heterocyst formation in *Anabaena* was shown to be slightly enhanced in nitrogen-depleted conditions under elevated exogenous Ca^2+^ concentrations ([Ca^2+^]_e_) up to 1–2mM, while concentrations above that have inhibitory and toxic effects on cell growth and health (Smith *et al.*, 1987; Singh and Mishra, 2014). Despite the apparent impact of Ca^2+^ on cell growth and development, changes in the amplitude and kinetics of Ca^2+^ signatures evoked upon nitrogen starvation and increased [Ca^2+^]_e_ were shown to be minor (Torrecilla *et al.*, 2004a).

Ca^2+^ effects have also been studied with respect to photosynthetic activity in cyanobacteria. Enhanced oxygen evolution resulted from increasing [Ca^2+^]_e_ in different cyanobacteria species, which may be linked to the involvement of Ca^2+^ in the photosystem II (PSII) oxygen evolving complex (Piccioni and Mauzerall, 1978). The link between Ca^2+^ and photosynthesis has received much more attention in plants (see Hochmal *et al.*, 2015 for a recent review), where it is known that the transition from light to darkness triggers a cytosolic Ca^2+^ signal that inhibits the enzymes of the Calvin–Benson–Bassham cycle. This observation of a dark-induced calcium signal has also been demonstrated in *Anabaena* (Torrecilla *et al.*, 2004b). This signal is most likely dependent on the redox state of photosynthetic electron transport chain components in plants as well as in cyanobacteria.

In this work, the impact of [Ca^2+^]_e_ on the induction of intracellular Ca^2+^ signalling in *Anabaena* has been explored through the analysis of gene expression, cell fitness and development. Here, it was found that [Ca^2+^]_e_ affects the C:N balance through short-term and long-term reprogramming of carbon- and nitrogen-specific metabolism, including up- and down-regulation of transporters, photosynthetic genes and transcription regulators. Changes in [Ca^2+^]_e_ impacted the accumulation of biomass and proteins, but had no significant effect on heterocyst differentiation in growth medium supplied with nitrate as a nitrogen source.

## Materials and methods

### Cultures and growth conditions

*Anabaena* sp. PCC 7120 cells were grown either in regular BG11 medium (Rippka *et al.*, 1979) (low Ca^2+^; 0.25mM CaCl_2_) or in BG11 medium plus additional CaCl_2_ (high Ca^2+^; 1mM CaCl_2_). Both media were supplemented with 10mM TES-KOH (pH 8.0). The cultures were cultivated under continuous low light (25–35 μmol photons m^−2^ s^−1^) at 30 °C and in 3% (vol/vol) CO_2_-containing air with mild shaking.

### Calcium shift experiment

*Anabaena* cells were grown under normal growth conditions in low and high Ca^2+^ media. After reaching OD_750_=1.0, the cells were centrifuged and the cell pellets resuspended to OD_750_=0.8 in either low or high Ca^2+^; for the Ca^2+^ treatments, pellets from high Ca^2+^ were resuspended in low Ca^2+^ medium (shifted from high to low) and pellets from low Ca^2+^ were resuspended in high Ca^2+^ medium (shifted from low to high). For the controls, pellets were resuspended in fresh medium with the same [Ca^2+^] (shifted from low to low or high to high). Afterwards, the cultures were again incubated under normal growth conditions. Over a time range of 24h (0, 1, 2, 4, 8, 16, 24h post-shift), samples were taken for the determination of total proteins, carbohydrates, and biomass. Data were collected from three independent Ca^2+^ shift experiments (*n*=3).

### Determination of the biomass

A 20ml culture volume was passed through a pre-washed, pre-dried and pre-weighed glass-fibre filter in a glass vacuum filtration apparatus and then washed with distilled water. Filters were dried at 60 °C for 24h, stored in a desiccator and then weighed.

### Determination of the protein content

A 0.2ml volume of culture was taken in triplicate, centrifuged and washed with distilled water, and the pellets were frozen. The total protein content was determined according to a modified Lowry procedure (Markwell *et al.*, 1978). OD_750_ was measured against a bovine serum albumin (BSA) standard curve with a Lambda 25 UV/VIS spectrometer (Perkin Elmer).

### Determination of total sugars content

A 1.0ml volume of culture was taken in triplicate, spun down and washed with distilled water. The total sugar content was analysed by the colorimetric method described by DuBois *et al.* (1956) after diluting the samples 1:1 with milliQ water.

### RNA isolation and next-generation sequencing

*Anabaena* cultures were shifted between low and high Ca^2+^ media as described above. Each of the four samples (low Ca^2+^ to high Ca^2+^, high Ca^2+^ to low Ca^2+^, and their controls) was represented by three independent biological replicates (*n*=3). One hour after the shift, the cultures were centrifuged (6000×*g*, 6min, RT) and the pellets immediately resuspended in 0.2ml RNA resuspension buffer (0.3M saccharose+10mM sodium acetate, pH 4.5) and 60 μl 0.25M ethylenediaminetetraacetic acid (EDTA) was added. A 0.3ml volume of lysis buffer (2% sodium dodecyl sulfate+10mM sodium acetate, pH 4.5) was added, followed by 0.5ml of phenol:chloroform:iso-amyl alcohol (P:C:IAA=25:24:1). Then the samples were vortexed thoroughly, incubated for 5min at 95 °C and centrifuged for 15min at 15000×*g*, 4 °C. The upper phase was collected, twice re-extracted with an equal volume of P:C:IAA and then extracted with C:IAA (24:1). The aqueous phase was supplemented with 10M lithium chloride to a concentration of 2.5M, and incubated overnight at –20 °C. The next day, the samples were centrifuged as before and the pellets washed with 1ml 70% ethanol by gentle mixing. After centrifugation, the RNA pellets were air-dried and resuspended in small volumes of milliQ water at 65 °C for 15min. RNA isolates were treated with DNase. RNA samples were submitted to the Turku Centre for Biotechnology (Turku, Finland) for RNA sequencing using a HiSeq 2000 (Illumina).

### RNAseq data analysis

RNAseq reads were aligned using the reference genome and annotations of *Nostoc* sp. PCC 7120, downloaded from Ensembl (EBI). Alignment was using the Tophat algorithm, and ORF calling and read-depth quantification were carried out using the open source analysis software Chipster (CSC, Finland). Significantly differentially expressed genes were identified using a false discovery rate (FDR: Benjamini–Hochberg) cutoff of 0.05. Gene descriptions were collected from Cyanobase (genome.microbedb.jp/cyanobase/) and KEGG (www.genome.jp/kegg/).

### Photosynthetic activity measurements

Oxygen evolution rate was measured using a Clark-type oxygen electrode (Hansatech Oxytherm) and quantum yield of PSII (Y(II)) was measured using a Dual-PAM-100 (Walz). For both, samples were dark-adapted for 5min. For the gross oxygen evolution rate in light, the rates of oxygen consumption obtained in darkness were added to oxygen evolution rates obtained in saturating light (intensity: 400 μmol photons m^−2^ s^−1^), and values were normalized to the chlorophyll content (chl a estimated in 90% methanol at OD_665_). For the determination of Y(II), samples were diluted to a chlorophyll concentration of 5 μg chl a ml^–1^ and the fluorescence parameters *F*_v_ and *F*_m_ determined according to Genty *et al.* (1989).

### Light and fluorescence microscopy

Bright-field and fluorescence images were taken using an AxioVert 200M fluorescence microscope (Zeiss) and a Wetzlar light microscope (Leitz) on ×40 magnification. From active *Anabaena* filaments, 1000–2000 cells were counted for each treatment and the heterocyst frequency calculated as a percentage of total cells counted.

### Determination of nitrogen concentration

Nitrate concentration of filtered media was determined spectrophotometrically using a Spectroquant Nitrate Test kit (Merck).

## Results

### Calcium induces both short-term and long-term changes in gene expression

**Fig. 1.**
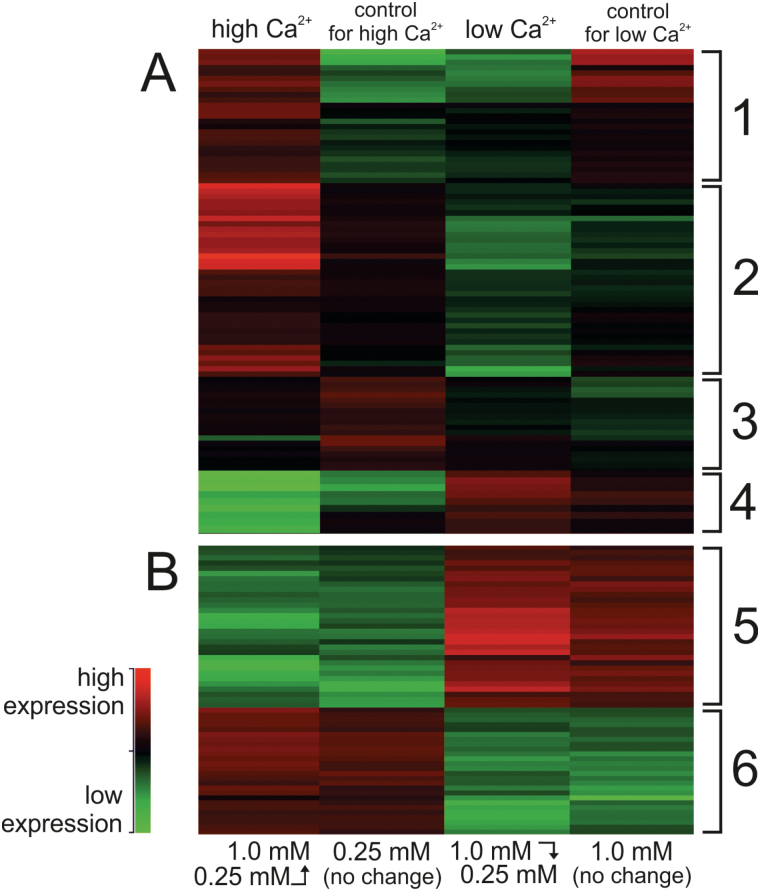
Hierarchically clustered heatmap of gene expression in *Anabaena* cells treated with high and low Ca^2+^, as well as in the controls for each. Columns show the absolute expression of significantly differentially expressed genes from triplicate samples (*n*=3) for each condition. Major clusters are indicated with numbers corresponding to Table 1 (and are discussed in text). (A) Genes responsive to 1h of Ca^2+^ treatment, called ‘short-term responsive genes’; (B) Genes resistant to short-term response but responsive to 3 days of Ca^2+^ treatment, called ‘long-term responsive genes’.

The response of *Anabaena* sp. PCC 7120 to changes in external Ca^2+^ conditions was studied by pre-growing cells to OD_750_=1.0 (over approximately 3 days) in either low or high Ca^2+^ medium (0.25 and 1mM CaCl_2_, respectively), and then replacing the medium with one of either the same Ca^2+^ concentration (acting as controls), or with an alternative Ca^2+^ concentration to create an up-shift (henceforth referred to as ‘high Ca^2+^’) or a down-shift (‘low Ca^2+^’) in [Ca^2+^]. The transcriptomic reaction of *Anabaena* cells to the change in [Ca^2+^] was analysed 1h post-shift by transcriptome sequencing. Gene expression in high Ca^2+^ and low Ca^2+^ were compared with the corresponding controls to identify genes that had a fold change (FC) ≥2 (log_2_ FC≥1) between treatment and control (FDR<0.05). Genes that were significantly differentially expressed after 1h in altered [Ca^2+^] were designated ‘short-term Ca^2+^-responsive genes’ (see Table 1). Genes were also identified that showed no significant short-term response to Ca^2+^ (i.e. were not differentially expressed between the shifts and their corresponding controls), but showed strong differential regulation in response to the Ca^2+^ conditions of the 3-day pre-culture. These genes were designated ‘long-term Ca^2+^-responsive genes’ (see Table 2). The absolute expression levels of genes in all four samples were hierarchically clustered to identify common expression profiles (Fig. 1).

**Table 1.** Differential expression of selected short-term responsive Ca^2+^-regulated genes

**Gene name**	**Accession**	**Description**	**log**_**2**_ **fold change in high Ca** ^**2+*a***^	**log**_**2**_ **fold change in low Ca** ^**2+**^	**FDR** ^***b***^	**Gene Cluster** (Fig. 1)
Bicarbonate import/metabolism						
*cmpA*	alr2877	Bicarbonate-binding protein	2.6	–2.2	0.000	1
*cmpB*	alr2878	Bicarbonate transport permease	2.5	–2.3	0.000	1
*cmcC*	alr2879	Bicarbonate transport, ATP-binding protein	2.0	–2.0	0.000	1
*cmpD*	alr2880	Bicarbonate transport, ATP-binding protein	2.3	–2.4	0.000	1
*bicA*	all1304	Low affinity bicarbonate Na^+^ symporter	1.8	–1.5	0.000	1
*NhaS3-like*	all1303	Na^+^:H^+^ antiporter	1.6	–1.4	0.000	1
*sbtB*	all2133	Bicarbonate Na^+^ symporter	1.1	–1.8	0.000	1
*sbtA*	all2134	Bicarbonate Na^+^ symporter	1.6	–2.1	0.000	1
*mrpB*	all1837	Na^+^:H^+^ antiporter subunit B	1.2	–0.4	0.000	1
*mrpA*	all1838	Na^+^:H^+^ antiporter subunit A	1.2	–0.5	0.001	1
*mnhG*	asl1839	Na^+^:H^+^ antiporter subunit G	1.3	–1.1	0.028	1
*mnhF*	asl1840	Na^+^:H^+^ antiporter subunit F	1.3	–1.0	0.025	1
*mnhE*	all1841	Na^+^:H^+^ antiporter subunit E	1.4	–0.7	0.785^*b*^	1
*mrpD*	all1842	Na^+^:H^+^ antiporter subunit D	1.3	–0.5	0.000	1
Nitrate import/metabolism						
*nirA*	alr0607	Ferredoxin:nitrite reductase	–1.5	0.5	0.010	4
*nrtA*	alr0608	Nitrate/nitrite transport substrate- binding protein	–1.6	0.5	0.011	4
*nrtB*	alr0609	Nitrate/nitrite transport permease	–1.3	0.5	0.032	4
*nrtC*	alr0610	Nitrate/nitrite transport ATP-binding protein	–1.2	0.7	0.000	4
*nrtD*	alr0611	Nitrate/nitrite transport ATP-binding protein	–1.0	0.9	0.005	4
*narB*	alr0612	Ferredoxin:nitrite reductase	–0.8	0.7	0.000	4
*cphB2*	all0571	Cyanophycinase, dipeptidase	1.1	0.1	0.001	2
Photosynthesis and carbon metabolism						
*psbAIII*	alr4592	PSII core protein	1.4	–0.4	0.000	2
*psbAIV*	all3572	PSII core protein	0.7	–0.2	0.002	2
*psbH*	all4050	PSII reaction centre protein	1.8	0.0	0.000	2
*psaE*	asl4098	PSI reaction centre subunit	2.0	–0.4	0.000	2
*fbaB*	all3735	Fructose-bisphosphate aldolase class I	1.0	–0.2	0.007	1
*glgB*	all0875	Alpha-glucanotransferase	1.4	–0.9	0.000	2
*asr3089*	asr3089	Transglycosylase-associated protein	1.2	–0.4	0.003	1
*alr1850*	alr1850	Transketolase	0.8	–0.5	0.032	1
Transcriptional regulators						
*rbcR1*	all0862	LysR-type regulator	1.0	–0.7	0.000	1
Stress-related genes						
*alr3199*	alr3199	Iron- and oxygen-binding HHE domain protein	1.7	–1.0	0.000	2
*all0457*	all0457	Low temperature-induced protein	1.1	–0.5	0.003	2
*all0458*	all0458	Low temperature-induced protein	1.5	–0.8	0.000	2
*all0459*	all0459	Ferritin-like protein, nutrient stress responsive	2.0	0.0	0.000	2
*alr5182*	alr5182	Short-chain dehydrogenase/reductase, desiccation responsive	1.4	–0.4	0.000	2
*alr0896*	alr0896	Unknown protein, induced by desiccation	1.3	–1.1	0.028	2
*asr1134*	asr1134	CsbD stress response protein	0.9	–0.4	0.015	2
*flv4*	all4446	Flavodiiron protein	0.7	–1.1	0.038	—
*flv2*	all4444	Flavodiiron protein	0.0	–1.2	0.735^*b*^	—
Bidirectional hydrogenase						
*alr0761*	alr0761	Peptidase domain-containing protein	–1.5	1.6	0.033	3
*hoxU*	alr0762	Bidirectional hydrogenase subunit	–0.4	0.0	0.227^*b*^	3
*alr0763*	alr0763	Hypothetical protein	–0.6	0.5	0.002	3
*hoxY*	alr0764	Bidirectional hydrogenase subunit	–0.9	0.7	0.000	3
*alr0765*	alr0765	Hypothetical protein	0.0	1.0	0.818	3
*alr0750*	alr0750	Hypothetical protein	–0.4	0.2	0.015	3
*hoxE*	alr0751	Bidirectional hydrogenase, diophorase subunit	–0.8	0.5	0.000	3
*hoxF*	alr0752	Bidirectional hydrogenase subunit	–0.8	0.6	0.136	3
Other proteins						
*alr0198*	alr0198	Hypothetical protein	1.1	–0.6	0.000	2
*alr0199*	alr0199	Hypothetical protein	1.1	–0.3	0.000	2
*alr3715*	alr3715	Hypothetical protein	1.7	–1.4	0.000	2

^*a*^ Genes with log_2_ expression fold change ≥1 are considered to be differentially expressed. However, in some cases genes of special interest with log_2_ <1 differential expression have been included.

^*b*^ Data with false discovery rate (FDR)>0.05 are included when a gene belongs to an operon with member genes that have statistically robust FDRs (<0.05).

**Table 2.** Selected genes with long-term expression response to Ca^2+^ All genes shown were <2 FC differentially expressed between the high or low Ca^2+^ shifts and their respective controls, and were >2 FC differentially expressed between both cultures originating from the high Ca^2+^ pre-culture (low Ca^2+^ and control for low Ca^2+^) and both of those originating from the low Ca^2+^ pre-culture (high Ca^2+^ and control for high Ca^2+^).

**Gene name**	**Accession**	**Description**	**FDR**	**Cluster** (Fig. 1)
Up-regulated in high calcium/down-regulated in low calcium				
*hglE*	alr5351	Heterocyst glycolipid synthase	0.007	5
*hglD*	alr5354	Heterocyst glycolipid synthase	0.014	5
*hglC*	alr5355	Heterocyst glycolipid synthase	0.009	5
*nifB*	all1440	Nitrogen fixation protein	0.008	5
*nifN*	all1437	Nitrogenase iron-molybdenum protein	0.023	5
*nifE*	all1438	Nitrogen iron/molybdenum cofactor biosynthesis	0.007	5
*nifK*	all1440	Nitrogenase iron-molybdenum protein	0.008	5
*nifH*	all1455	Nitrogenase iron protein	0.003	5
*nifU*	all1456	Nitrogen fixation protein	0.018	5
*nifS*	all1457	Nitrogenase cofactor synthesis protein	0.004	5
*chlN*	all5076	Protochlorophyllide reductase subunit	0.012	5
*chlL*	all5078	Protochlorophyllide reductase iron-sulfur ATP-binding protein	0.006	5
*clhB*	alr3441	Protochlorophyllide reductase	0.003	5
*chlH*	all4365	Protoporphyrin IX magnesium chelatase	0.014	5
*all0156*	all0156	Hypothetical protein	0.003	5
*all0157*	all0157	Hypothetical protein	0.002	5
*all0158*	all0158	Hypothetical protein	0.012	5
Down-regulated in high calcium/up-regulated in low calcium				
*rpl14*	all4205	50S ribosomal protein	0.004	6
*rpl29*	asl4207	50S ribosomal protein	0.028	6
*rpl16*	all4208	50S ribosomal protein	0.019	6
*rps3*	all4209	30S ribosomal protein	0.007	6
*rpl22*	all4210	50S ribosomal protein	0.006	6
*rps19*	asl4211	30S ribosomal protein	0.026	6
*rpl2*	all4212	50S ribosomal protein	0.039	6
*rpl23*	all4213	50S ribosomal protein	0.043	6
*rpl4*	all4214	50S ribosomal protein	0.003	6
*sigG*	alr3280	Group 3 sigma factor	0.002	6
*sigB2*	alr3800	Group 2 sigma factor	0.006	6

### Short-term Ca^2+^ shifts modulate the expression of carbon and nitrogen transport and photosynthesis genes

Genes related to bicarbonate (HCO_3_^–^) uptake were among the most strongly affected short-term Ca^2+^-responsive genes. All eight components of the three known HCO_3_^–^ transporters in *Anabaena*, which are encoded on three separate operons, underwent 4–6.5 FC up-regulation (log_2_ FC of 2–2.6) in high Ca^2+^ shift. Conversely, the same genes were strongly down-regulated in low Ca^2+^ shift (Table 1). According to their expression profile, the HCO_3_^−^ transporter genes populate Cluster 1 (Fig. 1a), which also includes all subunits of the mrp Na^+^:H^+^ antiporter complex and the LysR-type transcriptional regulator rbcR1 (*all0862*). Additionally, several Calvin cycle and metabolic enzymes, including fructose bisphosphate aldolase, transketolase and the petF ferredoxin (*all4148*), shared the Cluster 1 expression profile.

Cluster 2 genes were strongly up-regulated in the short-term response to high Ca^2+^ and moderately down-regulated by low Ca^2+^. Several photosynthetic subunits were encoded in Cluster 2, as well as stress-responsive genes including a low-temperature induced operon (*all0457–all0459*; Sato *et al.*, 2012), an oxygen-binding HHE domain protein (*alr3199*), and a desiccation-responsive dehydrogenase (*alr5182*; Katoh, 2012). The cyanophycinase *cphB2* (*all0571*) was also strongly up-regulated in high Ca^2+^.

Operons encoding the subunits of the bidirectional ‘hox’ hydrogenase were down-regulated in response to high Ca^2+^ and up-regulated in low Ca^2+^ (Table 1). These genes were included in expression Cluster 3 together with *nifJ* and *hoxR*, which had <2FC differential expression but were nonetheless clearly down-regulated in high Ca^2+^ shift and up-regulated in low Ca^2+^ in comparison with the controls.

The *nir* operon that encodes the nitrate/nitrite transporter complex was down-regulated in high Ca^2+^, and up-regulated in low Ca^2+^ shift (Table 1), although these genes had low expression in both high Ca^2+^ and its control culture, and high expression in both low Ca^2+^ and its control (see Fig. 1A). This suggests that expression of the *nir* operon and other Cluster 4 genes (Fig. 1A) was predominantly influenced by the conditions of the pre-culture, which were common for each shift and its control; however, these genes also demonstrated a short-term response to Ca^2+^ that led to differential regulation 1h after the shift.

### Calcium has a long-term effect on genes encoding nitrogen metabolism

Genes that were induced by long-term high Ca^2+^ conditions and repressed by long-term low Ca^2+^ included heterocyst glycolipid synthesis (*hgl*) genes and several members of the *nif* operon that encode the heterocyst-specific nitrogen fixing machinery in *Anabaena* (Table 2). Subunits of the light-independent protochlorophyllide reductase (DPOR) were also among long-term Ca^2+^ up-regulated genes that are shown in Cluster 5 (Fig. 1B). Cluster 6 genes that underwent significant repression and induction by long-term high and low Ca^2+^ conditions, respectively, encoded nine proteins of the 50S and 30S ribosome, as well as the group 2 sigma factor *sigB2* (also called *sigE*) and ECF sigma factor *sigG* (Table 2).

### Effects of calcium on *Anabaena* growth and biomass composition

**Fig. 2.**
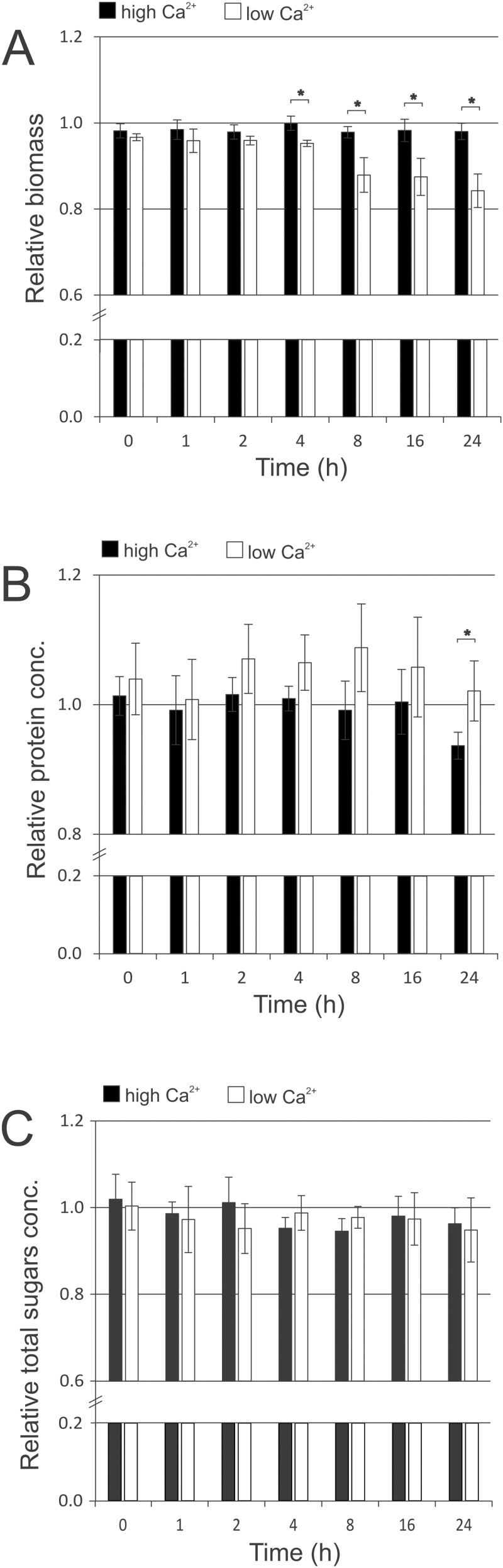
Biomass (A), total protein (B) and total sugars (C) composition of *Anabaena* sp. PCC 7120 cells shifted to high Ca^2+^ (0.25mM to 1mM) or low Ca^2+^ (1mM to 0.25mM) relative to the controls. Data presented are averages of three biological replicates (*n*=3). Error bars show standard deviations. Significant differences between high Ca^2+^ and low Ca^2+^ samples are indicated with asterisks (*t*-test *P*<0.05).

Biomass accumulation and total protein and total sugars content of *Anabaena* cells were followed for 24h after cultures were shifted to the same high and low Ca^2+^ media and using the same control samples as described above. *Anabaena* cells shifted to low Ca^2+^ resulted in a biomass penalty. These cells showed a decreasing trend in biomass accumulation throughout the experiment in comparison with the control, from a ratio of approximately 1 early in the experiment to less than 0.9 at 24h (Fig. 2A). This was in contrast to no relative change in biomass observed for cells shifted to high Ca^2+^.

Whilst the low Ca^2+^ medium penalized biomass accumulation over time, the relative protein composition of this biomass was higher than that of control cells throughout the 24h growth period (Fig. 2B). The relative protein composition of cells in low Ca^2+^ reached the highest ratio at 8h after the shift in Ca^2+^ concentration, which correlates with the observed up-regulation of nitrate transport genes. There was less variation in the relative protein composition of cells in high Ca^2+^, which remained fairly stable at around 1 until 16h, but was significantly lower than the low Ca^2+^ shift at 24h.

The shift in Ca^2+^ concentration had no observable effect on the total sugars content of *Anabaena* cells in the current experiment, with the relative concentrations remaining close to 1 and similar between high and low Ca^2+^ shifts throughout (Fig. 2C).

### Effects of calcium on the photosynthetic activity of *Anabaena*

**Fig. 3.**
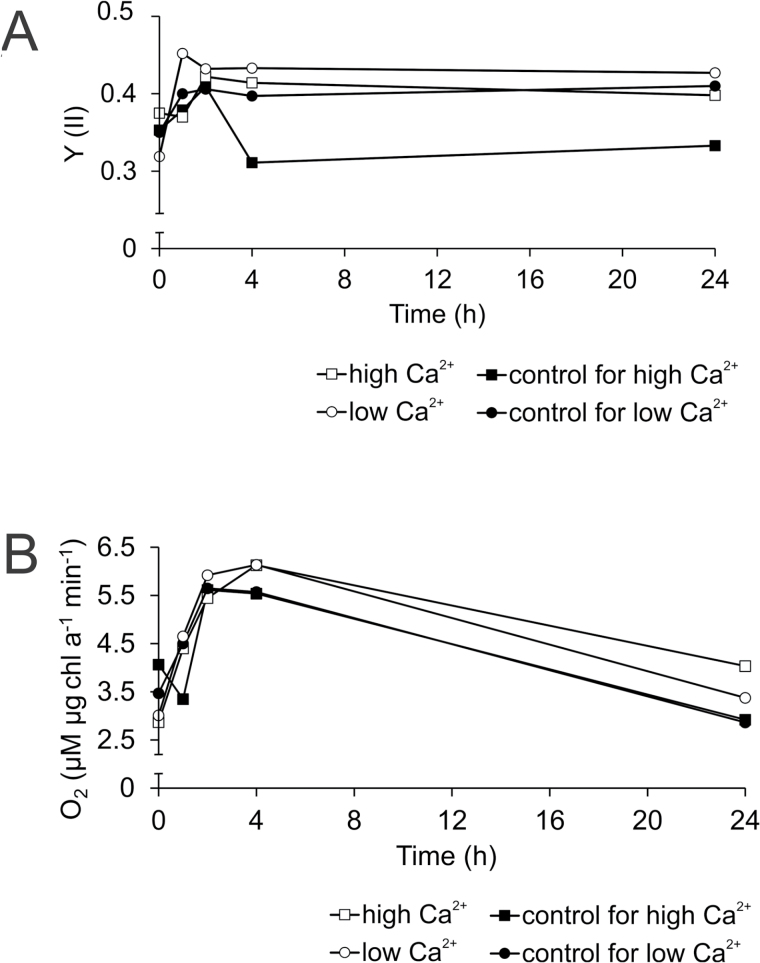
Photosynthetic activity of *Anabaena* sp. PCC 7120 cells shifted to high Ca^2+^ (0.25mM to 1mM, open squares) or to low Ca^2+^ (1mM to 0.25mM, open circles) and of the controls (for high Ca^2+^, closed squares; and for low Ca^2+^, closed circles). Dual-PAM measurements of PSII yield (A) and oxygen evolution rates (B) shown here are from single experiments (*n*=1) that represent photosynthetic response to change in [Ca^2+^]_e_. Lines connecting data points are included to aid visualization of the series.

Fluorescence kinetics of PSII and oxygen evolution in *Anabaena* cultures shifted to low and high Ca^2+^ conditions showed an increase in PSII yield in all cultures in the first 2h after the shift (Fig. 3A). After 2h, the PSII yield stabilized at 0.35–0.38 for all cells, except in the low Ca^2+^ control cells. These cells experienced a further 28% drop in PSII yield from 2 to 4h and then stabilized until the 24h time point. Consequently, a relatively higher photosynthetic yield was observed after 2h for the *Anabaena* cells shifted to high Ca^2+^. Similarly, a rapid increase in gross oxygen evolution was observed for all cells over the first 2h, resulting in an approximate doubling of recorded values followed by a slow decline throughout the remainder of the experiment (Fig. 3B).

### Changes in calcium concentration have no effect on heterocyst differentiation in *Anabaena* in the presence of combined nitrogen

**Fig. 4.**
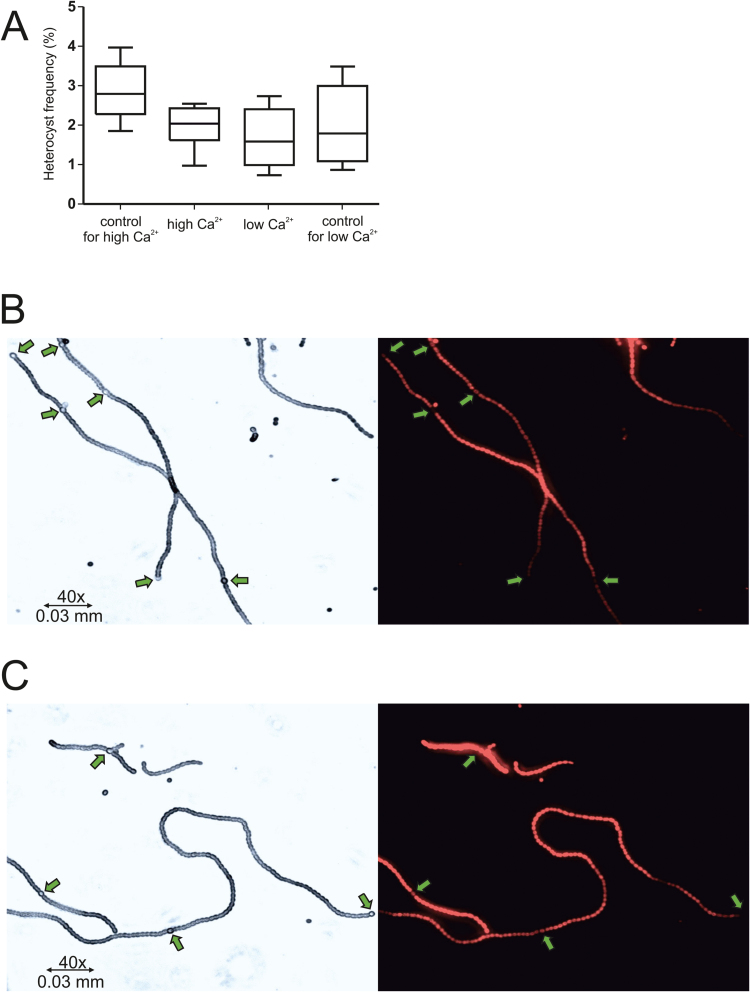
Box and whisker plot showing the heterocyst frequency rates (A) and representative bright field and fluorescence micrographs of *Anabaena* sp. PCC 7120 shifted to high Ca^2+^ (0.25mM to 1mM; B) or to low Ca^2+^ (1mM to 0.25mM; C) and corresponding controls (A only) 2 days post-shift. Arrows indicate nitrogen-fixing heterocysts.

Heterocyst frequency was evaluated at 10h, 20h and 2 d after the change in Ca^2+^ conditions, and was determined not to be statistically different between low and high Ca^2+^ shifted cells (*t*-test, 0.95 confidence; *P*=0.32). Heterocyst frequency data are shown in Fig. 4 and Supplementary Table S1 at *JXB* online. To investigate this further, the combined nitrogen levels were determined in the filtered media taken from all time points, and shown to be approximately 200mg L^−1^ NO_3_^−^-N for both high and low Ca^2+^ media.

## Discussion

### External calcium affects the gene expression and primary metabolism of *Anabaena*

The role of Ca^2+^ ions in signal transduction is widely recognized in eukaryotic organisms (Clapham, 1995) and has increasingly become a focus of study in prokaryotes in recent years (Onek and Smith, 1992; Norris *et al.*, 1996; Michiels *et al.*, 2002; Dominguez *et al.*, 2015). The maintenance of very low basal intracellular Ca^2+^ concentrations by active efflux from the cell and through the activity of Ca^2+^-binding proteins (Gangola and Rosen, 1987; Carafoli *et al.*, 2001; Batistic and Kudla, 2012; Stael *et al.*, 2012b; Dominguez *et al.*, 2015) allows increases in cytosolic Ca^2+^ to have a potent signalling effect. The intracellular Ca^2+^ concentration of the filamentous cyanobacterium *Anabaena* was shown to rise transiently as a result of increased external Ca^2+^, as well as in response to temperature and osmotic stress (Torrecilla *et al.*, 2000, 2001). However, the major signalling role of Ca^2+^ is associated with the differentiation of vegetative cells into nitrogen-fixing heterocyst cells under nitrogen-limited conditions, through the activity of several regulator proteins that are sensitive to the metabolic status of the cell (Torrecilla *et al.*, 2004a; Zhao *et al.*, 2005; Shi *et al.*, 2006; see Kumar *et al.*, 2010 for a review). In this work, a 4-fold increase in external Ca^2+^ concentration caused rapid and strong up-regulation of the HCO_3_^–^ transporters Cmp (also called BCT1), Bic and Sbt, as well as the Mrp operon that is involved in supplying Na^+^ to the latter two HCO_3_^−^ symporters. These transporters are components of the carbon-concentrating mechanism (CCM) that also includes CO_2_ transport machinery in the plasma and thylakoid membranes (Shibata *et al.*, 2002; Badger *et al.*, 2002; Badger and Price, 2003). The opposite transcription response of HCO_3_^−^ transporters occurred under a 4-fold decrease in Ca^2+^. In the unicellular cyanobacterium *Synechocystis* sp. PCC 7002, Cmp and Sbt are expressed in response to limiting C_i_ conditions (Woodger *et al.*, 2007); however, cultures used in the current study were grown under 3% CO_2_ and did not experience C_i_ limitation. Neither was this caused by any change in media pH, which measured 7.5–8.0 throughout the experiment and was identical in all cultures. Low C_i_-induced expression of the Cmp operon in *Anabaena* is controlled by the LysR-type regulator All0862, which is itself up-regulated in response to low nitrogen (Lopez-Igual *et al.*, 2012). In this work, the 2FC up-regulated expression of *all0862* observed in high Ca^2+^ may explain up-regulation of the Cmp transporter, although these cultures were not under nitrogen deprivation (discussed below). Notably, translocation of extracellular HCO_3_^−^ by the cmpA subunit requires co-binding of Ca^2+^ and HCO_3_^−^ ions to cmpA (Koropatkin *et al.*, 2007). It has not been established whether these Ca^2+^ translocation cofactors are released into the cell; however, in this way increases in [Ca^2+^]_e_ could rapidly translate to an internal signal through increasing the intracellular concentration of Cmp-transported HCO_3_^−^, rather than directly through increased [Ca^2+^]_i_. A transient rise in [Ca^2+^]_i_ in response to increased [Ca^2+^]_e_ was shown to be rapidly reversed in *Anabaena* (Torrecilla *et al.* 2000), whereas an increase in HCO_3_^−^ could induce a more long-term signal through metabolic intermediates such as 2-oxoglutarate (2-OG). Carbohydrate measurements in this study showed no difference between high and low Ca^2+^ that would reflect the up- and down-regulation of HCO_3_^−^ transporters. Rapid induction of several photosynthetic genes under high Ca^2+^ may be connected with the increased uptake of HCO_3_^−^, which is converted to CO_2_ for photo-assimilation (reviewed in Burnap *et al.*, 2015). However, analysis of O_2_ evolution and PSII yield in this work did not provide evidence of an increased rate of photosynthesis under high Ca^2+^.

The concentration of Ca^2+^ demonstrated a short-term effect on the expression of the *nirA* operon that encodes the nitrate assimilation machinery, which was induced in low Ca^2+^ and repressed in high Ca^2+^. This correlates with the higher relative protein composition of cells shifted to low Ca^2+^ (Fig. 2B), and was the opposite trend to that observed for HCO_3_^−^ transport and photosynthesis genes. Expression of the *nirA* operon requires a lack of ammonium and is increased by both nitrate levels (indicated by extracellular or intracellularly generated nitrite) and by 2-OG, a metabolite which represents cellular C:N levels. These indicators respectively activate the ntcB and ntcA transcriptional activators of *nirA* (Ohashi, 2011). NtcA, considered the global nitrogen regulator of cyanobacteria (Herrero *et al.*, 2001), is crucial for the expression of the *nirA* operon across all cyanobacteria (Maeda *et al.*, 1998). NtcA activity is also increased by PipX, which is sequestered by the signalling protein PII under low 2-OG (Espinosa *et al.*, 2006). Interestingly, modification of the PII protein was suggested to be influenced by Ca^2+^ (Zhao *et al.*, 2005), though evidence for this is lacking. PII is involved in cyanophycin distribution between vegetative cells and heterocysts (Laurent *et al.*, 2004) and the up-regulation of cyanophycinase (*cphB2*) was observed under high Ca^2+^ conditions in this study (Table 1). Thus it appears likely that Ca^2+^ affects the sensing of intracellular nitrogen availability, possibly through interaction with PII, resulting in the differential regulation of the *nirA* operon.

### *Anabaena* heterocyst differentiation is not affected by increased extracellular calcium under nitrate-replete conditions

It is interesting that the heterocyst-specific nitrogen fixing (*nif*) genes are up-regulated by high Ca^2+^ in media replete with nitrate (Fig. 1, Cluster 5), whilst the heterocyst frequency did not differ significantly between high and low Ca^2+^ conditions. In *Anabaena* sp. PCC 7120, nitrogenase is protected from oxygen-induced inactivation by being spatially segregated in specialized heterocyst cells where *nif* expression has been reported to occur late during heterocyst development, at 12–24h after nitrogen deprivation (Elhai and Wolk, 1990; Golden *et al.*, 1991; Herrero *et al.*, 2013). Previous work has demonstrated that intracellular Ca^2+^ is directly involved in heterocyst differentiation (Zhao *et al.*, 2005; Shi *et al.*, 2006), but this has generally been studied under conditions of combined nitrogen deprivation. Under such conditions the levels of 2-OG are increased, and NtcA is activated and binds to the promoter of the cyanobacterial Ca^2+^-binding protein (CcbP), decreasing *ccbP* expression and increasing free [Ca^2+^]_i_. Free [Ca^2+^]_i_ is also increased in heterocysts through the degradation of CcbP by HetR, the master regulator of heterocyst development. Collectively, the [Ca^2+^]_i_ of heterocyst cells reaches concentrations severalfold higher than that of vegetative cells (Zhao *et al.*, 2005; Shi *et al.*, 2006). Unlike the effect on *nif* expression, significant effects of [Ca^2+^]_e_ were not detected in this study for *HetR* or *NtcA* expression, which have been reported to occur in the early and intermediate stages of heterocyst development (Herrero *et al.*, 2013). Nor was a Ca^2+^-mediated differential expression of *PatS* or *HetN* observed, both of which are involved in the negative regulation of heterocyst differentiation (Orozco *et al.*, 2006). However, the expression level of these genes does not necessarily represent their activity. On the other hand, the effects of high [Ca^2+^]_e_ in the nitrate-replete conditions of the current work appear to act only on specific functional genes, including the heterocyst glycolipid synthase (*hgl*) gene cluster (Table 2), coding for a glycolipid layer deposited between the outer membrane and polysaccharide layer and required for preventing diffusion of oxygen into the heterocyst (Fan *et al.*, 2005). This may indicate the structural and functional importance of heterocysts in maintaining a high [Ca^2+^]_i_ once differentiation has actually occurred.

Whilst *nif* and *hgl* genes demonstrated long term responses of up-regulation in high Ca^2+^ and down-regulation in low Ca^2+^, sigma factors *sigB2* and *sigG*, which are both involved in, but not individually required for, heterocyst development (Brahamsha and Haselkorn, 1992; Khudyakov and Golden, 2001; Ehira and Miyazaki, 2015), demonstrated opposite long-term responses to Ca^2+^. Additionally, *nrra*, another gene involved in early heterocyst development (Ehira and Ohmori, 2006), was found to be strongly (but not significantly; FDR>0.05) down-regulated in high Ca^2+^.

### Ca^2+^ influences C:N balance in *Anabaena*

This study has found that changes in Ca^2+^ concentrations lead to opposite regulation of C and N assimilation pathways that nonetheless do not lead to major changes in cellular biochemistry or development. This suggests that Ca^2+^ signalling in *Anabaena* may have a role in fine-tuning the C:N balance of cells for optimal growth under fluctuating environmental conditions. One possible mechanism for this may be through modulating the activity of a transcriptional regulator. Given the central role of the NtcA regulator, which generally activates, but can also repress, genes involved in management of the C:N balance in *Anabaena* (Herrero *et al.*, 2004), the expression of approximately 40 known NtcA-regulated genes (Picossi *et al.*, 2014) was tested using RNAseq data from the current study. This analysis revealed no clear correlation between NtcA regulation and Ca^2+^ treatment, although, several of these genes were significantly differentially expressed under shifted [Ca^2+^]_e_ (see Supplementary Figure S1 at *JXB* online).

Considering the tight regulation of [Ca^2+^]_i_ in *Anabaena* (Torrecilla *et al.* 2000), the up-regulation of nitrogen metabolism- and heterocyst-related genes as a long-term response to increased [Ca^2+^]_e_ may most likely be a metabolic adjustment in response to the up-regulation of carbon metabolism in the short term. In this case, the observed increase in *nif* expression may be induced through increased 2-OG levels as a consequence of increase of Cmp-transported HCO_3_^−^. Nonetheless, the possibility that even trace quantities of [Ca^2+^]_i_ can influence gene expression through Ca^2+^-binding transcription regulators, despite strict [Ca^2+^]_i_ maintenance, should not be dismissed.

### Perspective

This work shows that Ca^2+^ operates as a secondary messenger that affects the primary cellular metabolism of *Anabaena* under conditions replete in both combined-nitrogen and inorganic carbon. Transcriptomics data revealed distinctly and strikingly opposite trends in regulation of nitrogen-related and carbon-related processes in response to Ca^2+^. Given the elaborate and sensitive network of regulation of C:N balance in cyanobacteria, it is tempting to speculate that Ca^2+^ has a prominent role in the initial stages of C:N balance adjustment, through regulation of cellular nutrient levels, photosynthesis and/or gene activation. This short-term effect of Ca^2+^ likely prompts a cascade of gene expression to rebalance the C:N homeostasis of the cell. However, heterocyst differentiation and nitrogen fixation in *Anabaena* appear to require convergence of multiple signalling pathways in addition to Ca^2+^, including those instigated by genuine combined nitrogen deficiency. It will be most valuable to establish the identities of the cellular factors responsible for transforming the Ca^2+^ signal to gene expression in *Anabaena*, especially metabolic intermediates such as 2-OG and protein receptors of Ca^2+^.

## Supplementary data

Supplementary data are available at *JXB* online.

Figure S1. Clustered heatmap showing the absolute expression of genes shown to interact with the transcriptional regulator NtcA (Picossi *et al.* 2014) in response to changes in [Ca^2+^] in the expression data of the current study.

Table S1. Heterocyst counts in Ca^2+^ shift experiments.

Supplementary Data
