# Supplementary material for: Calcium impacts carbon and nitrogen balance in the filamentous cyanobacterium Anabaena sp. PCC 7120
Source: J Exp Bot. 2016 Mar 24;67(13):3997–4008. doi: 10.1093/jxb/erw112 (PMC4915528; doi:10.1093/jxb/erw112)
Supplement: Supplementary Data [file supp_67_13_3997__index.html]

Calcium impacts carbon and nitrogen balance in the filamentous cyanobacterium Anabaena sp. PCC 7120 — Calcium impacts carbon and nitrogen balance in the filamentous cyanobacterium Anabaena sp. PCC 7120 — Supplementary Data 

# Calcium impacts carbon and nitrogen balance in the filamentous cyanobacterium *Anabaena* sp. PCC 7120

## Supplementary Data

Data files

- Supplementary\_figure\_S1.pdf - Supplementary Data
- Supplementary\_Table\_S1.xlsx - Supplementary Data
