# Supplementary material for: Calcium impacts carbon and nitrogen balance in the filamentous cyanobacterium Anabaena sp. PCC 7120
Source: J Exp Bot. 2016 Mar 24;67(13):3997–4008. doi: 10.1093/jxb/erw112 (PMC4915528; doi:10.1093/jxb/erw112)

Supplementary Figure

Clustered heatmap showing the absolute expression of genes shown to interact with the transcrptional regulator NtcA (Picossi et al. 2014) in response to changes in [Ca2+] in the expression data of the current study. Entities with high expression are red, those with low expression are green. Black indicates intermediate expression

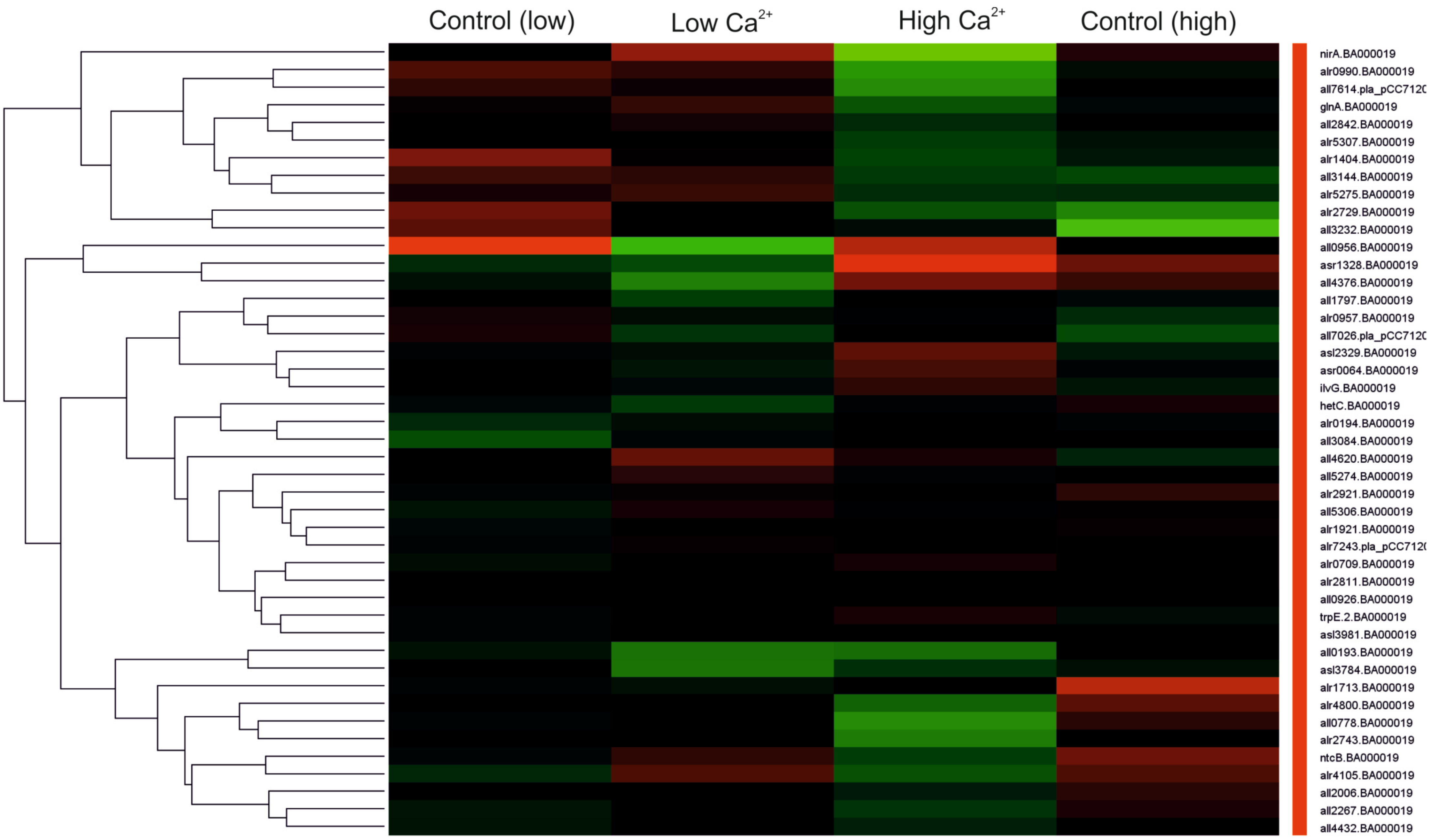

Supplement: Supplementary Data [file supp_erw112_Supplementary_figure_S1.pdf]
